# Musculoskeletal Disorders Associated with Occupational Driving: A Systematic Review Spanning 2006–2021

**DOI:** 10.3390/ijerph19116837

**Published:** 2022-06-02

**Authors:** Olivia Pickard, Peta Burton, Hayato Yamada, Ben Schram, Elisa F. D. Canetti, Robin Orr

**Affiliations:** 1 Faculty of Health Sciences and Medicine, Bond University, Robina 4226, Australia; olivia.pickard@student.bond.edu.au (O.P.); peta.burton@student.bond.edu.au (P.B.); hayatokingsley.yamada@student.bond.edu.au (H.Y.); bschram@bond.edu.au (B.S.); ecanetti@bond.edu.au (E.F.D.C.); 2 Tactical Research Unit, Bond University, Robina 4226, Australia

**Keywords:** vibration, lower back pain, vehicle ergonomics, posture, truck, bus, taxi

## Abstract

Several occupations require workers to spend long periods of time driving road vehicles. This occupational task is associated with musculoskeletal disorders. The purpose of this review was to collate, synthesize, and analyze research reporting on musculoskeletal disorders associated with occupational driving, in order to develop a volume of evidence to inform occupational disorder mitigation strategies. A systematic search of academic databases (PubMed, EBSCO host, CINAHL, SPORTDiscus, and Web of Science) was performed using key search terms. Eligible studies were critically appraised using the Joanna Briggs Institute critical appraisal checklists. A Cohen’s kappa analysis was used to determine interrater agreement between appraisers. Of the 18,254 identified studies, 25 studies were selected and appraised. The mean critical appraisal score is 69% (range 38–100%), with a fair level of agreement (k = 0.332). The studies report that musculoskeletal disorders, most commonly lower back pain, is of concern in this population, particularly in truck, bus, and taxi drivers. Risk factors for these occupations include long hours in a sitting position, years in the profession, vehicle ergonomics, and vibration.

## 1. Introduction

Driving a vehicle is a common occupational task. From maintaining daily public transport demands to channeling cargo efficiently via vast road networks across the globe, millions of people rely on those who dedicate their careers to a life behind the wheel. Industrialization and large migration rates are the foundations fostering the growth of the transport industry [1]. Examples of this can be seen in countries such as Canada, where truck driving is the second most common occupation [2]; Hong Kong, where double decker buses are a major means of transport due to heavily congested roads [3]; and Korea, which saw 95,488 people engaged in bus driving as an occupation in 2011 [4]. These countries, and many others, also show a high prevalence of work-related musculoskeletal (MSK) disorders [1,2].

The task of driving itself is not perceived to have a high physical workload, however, the prolonged posture maintained while driving for long periods can take a physical toll on the body [5,6]. A stable driving posture requires the neck, back, shoulder, and arm muscles to maintain static muscular tension over a sustained period, producing localized muscular fatigue, aches, and pains [6]. Prolonged continuous sitting and long working hours make drivers more susceptible to abnormal or poor postures, which, in turn, can be influenced by ergonomic mismatches of the driving area, including seat comfort and design, causing undue stress on the spine [4,5,6,7,8,9]. Long term exposure to whole-body vibrations, jerky and repetitive movements, and prolonged sedentary lifestyles, present as key aggravating factors, which increase with years as an occupational driver [5,10]. Other contributing factors include increased traffic congestion, poor quality roads, increased job demands, and time constraints, all of which pose further risks to the health of occupational drivers [6,11]. Thus, it is not surprising that occupational driving, associated with long driving hours and sustained postures, serves as a risk factor for MSK disorders [12].

There are many different occupations that come under the broad term of occupational driving; these are generally based on the vehicles being driven and include, but are not limited to, truck drivers, taxi drivers, and bus drivers. Truck drivers present with illness and injury incidence rates higher than other driving occupations, while long-haul truck drivers experience more severe ergonomic strain, leading to injury and MSK disorders [13,14]. Taxi drivers are also reported to be at high risk of MSK disorders. For example, in the USA, taxi drivers are found to have a higher incidence (73.2 per 10,000) when compared to workers in non-driving occupations (34.3 per 10,000) [15]. Conversely, bus drivers are found to present with a high prevalence of lower back pain (LBP) (59% of 147 drivers), when compared to motor car (26%) and truck (16%) drivers [16].

LBP is a common work-related musculoskeletal complaint, and is considered one of the leading causes of activity limitation, disability, inability to work, and absenteeism in the workplace [5,8]. Almost every individual is expected to experience LBP during their working life, thought to be related to abnormal and/or persistent working postures [9]. As such, it is not unexpected that LBP has a prevalence rate of 84% across an occupational driver’s lifetime [1], or that, in general, occupational drivers are at frequent or high risk of LBP [7,17,18,19]. However, LBP is not the only MSK disorder sustained by drivers, with other disorders suffered by occupational drivers including neck [20,21,22], shoulder [21,22,23], knee [11], and foot [24] pain. Noting this range of musculoskeletal injury sites, various mechanisms are associated with causing these work-related MSK disorders.

Several different risk factors associated with causing work-related MSK disorders are associated with occupational driving [11,25,26,27,28,29]. For example, prolonged hours spent in a seated position while driving is associated with causing knee pain [11]. Lower body vibrations are repeatedly identified to increase the likelihood of LBP [11,22,25,26,27,28,29]. A longitudinal study of 202 drivers (garbage truck, garbage compactor, and bus) found occupational drivers are at high-risk of developing LBP, due to long-term driving and exposure to whole-body vibrations [27]. Further supporting this association between increased driving time and injury risk, are findings that older occupational drivers (65 years) have a greater risk of work-related MSK disorders when compared to younger drivers (25 years), due to longer periods of whole-body vibrations [29]. Irregularities on the road surface, or speed bumps, and the oscillation of the seat whilst the car accelerates are also considered to contribute to MSK disorders [11].

Noting the importance of driving as an occupation, it is imperative to consider the subsequent impact of occupational driving on MSK disorders and injury risk, as well identifying the associated causes and mechanisms of these disorders if risk mitigation strategies are to be developed. Therefore, the aim of this systematic review was to collect, critically appraise, and synthesize the findings of research investigating work-related MSK disorders associated with occupational driving and concomitant contributing factors to these injuries, in order to develop a volume of evidence to inform mitigation strategies.

## 2. Methods

A systematic review was conducted following the Preferred Reporting Items for Systematic reviews (PRISMA) guidelines [30]. The project and protocol for this systematic review were registered with the Open Science Framework on the 11 November 2021 (https://osf.io/b4uz6/) [31], prior to the database search conducted later that same day.

### 2.1. Search Methods

A systematic search of key databases (PubMed, Elton B. Stephens Company (EBSCO), (Birmingham, AL, USA) host, the Cumulative Index to Nursing and Allied Health Literature (CINAHL), SPORTDiscus, and Web of Science) was conducted in November 2021, using dedicated search terms. The search terms were selected based on key concepts identified during an initial rapid literature search. To help identify and refine the search strategies, 10 studies, broadly reviewed and considered to be within the scope of the review, were identified and their PubMed Identifier (PMIDs) numbers were input into a SR-accelerator search term refiner [32]. Following the use of the refiner tool [32], search terms were finalized, encompassing three key themes derived from the population, exposure, and outcome (PEO), whereby the population was occupational drivers, the exposure was task associated with occupational driving, and the outcome was injury. These themes were ‘Driver’, ‘Musculoskeletal’, and ‘Injuries’. For this review, injury was defined as any musculoskeletal disorder that arose from performing work tasks, with the exception of those caused by a motor vehicle accident. While these injuries were typically chronic in nature (e.g., lower back pain), acute injuries, if associated with an occupational task (e.g., ligament sprain of the knee exiting a vehicle), were included. The established PubMed search string was inserted into a Polyglot Search tool [33] to formulate the search string specific to each database. Filters were used, where available, in the search databases, to further optimize the search and ensure only studies relevant to this review were collected. Filters included: human, English, and year (2006–2021). For the Web of Science database, exclusions for surgery, neurology, and oncology were used. The full search term string for each database is provided in Table 1.

### 2.2. Study Selection and Eligibility Criteria

Following the data search, results were imported into Endnote 20.2 (Clarivate Analytics, Philadelphia, PA, USA), and duplicates were removed. The remaining studies were screened by title and abstract for relevance, and any studies clearly not of relevance to this review were excluded. The remaining studies were then retrieved and assessed against the eligibility criteria (Table 2). Studies that met the inclusion criteria but did not meet the exclusion criteria formed the final studies for review. Excluded studies, and their reasons for exclusion, were documented. This process was conducted by two reviewers independently (OP and PB).

### 2.3. Data Extraction and Synthesis

After finalizing the study selection, key data of studies informing this review were extracted under the main areas of relevance, including study authors and year, country of study, participants, vehicle type, setting, outcome measures used, and the key findings from each study. Two reviewers (HY and PB) independently extracted data from one study to create a data extraction table template. Where disagreements in approaches were found, consensus was reached by a third reviewer (OP). Risk factors were extracted, where possible, as well as prevalence of injury or musculoskeletal disorder data. All tabulated data were independently extracted by two reviewers (HY and PB.) Once data were extracted and tabulated, naturally emerging themes were used to synthesize the data.

### 2.4. Quality Assessment

Studies forming this review were then critically appraised using the Joanna Briggs Institute (JBI) critical appraisal checklist for analytical cross-sectional studies and quasi-experimental studies [37]. The JBI tool was comprised of eight questions, which could be answered as ‘Yes’, ‘No’, or ‘Unclear’. To allow for result visualization, a scoring system was applied, whereby a ‘Yes’ answer equaled one point, and an answer of ‘No’ or ‘Unclear’ equaled zero points. All studies were independently assessed by two reviewers (PB and HY). A Cohen’s kappa analysis was used to determine the level of agreement between the two reviewing authors by another reviewer (RO), and graded based on the system proposed by Landis [38]. Where there was a difference in scores between the two authors, the studies were reassessed by a third reviewer (OP) to determine the final study score. The final study critical appraisal score (CAS) was converted to a percentage by dividing the final possible score of each study from the maximal possible score allowed by the checklist, and multiplying the result by 100. The methodological quality of studies which scored less than 45.4% were considered ‘poor’, considered ‘fair’ if they scored between 45.4% and 61%, and ‘good’ if they scored over 61% [39].

## 3. Results

### 3.1. Search Strategy and Sources of Evidence 

**Figure 1:**
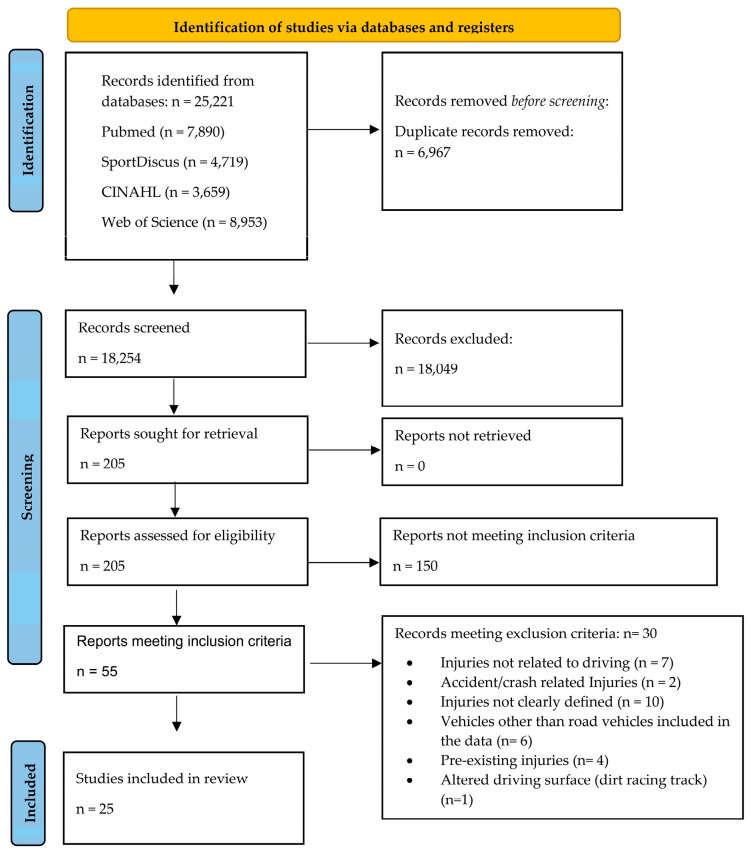
PRISMA flow diagram detailing the identification, screening, and final inclusion of studies.

A summary of the search results is provided in the PRISMA flow diagram (Figure 1) [30]. Of the initial 25,221 identified studies, 18,254 studies were screened by title and abstract, with 18,199 studies excluded as not being of relevance for the current review (e.g., study investigated injuries associated with driving for golf [40]). Studies were then retrieved and assessed for eligibility against the inclusion and exclusion criteria (n = 55). Studies meeting the exclusion criteria (n = 30), and their reasons for exclusion, are detailed in Supplemental Table S1. A final 25 studies were used to inform this literature review.

### 3.2. Critical Appraisal Results 

The mean critical appraisal score, based on the JBI checklist [37], is 69 ± 16%. Three studies receive 38% [4,5,12], the lowest score, while one study receives 100%, the maximum score [6]. The level of agreement (k = 0.335), as determined through a Kappa analysis, is considered “fair” [38]. Questions 1, 2, and 5 from the JBI checklist for analytical cross-sectional studies [37] show the least agreement. The focus of these questions is on inclusion criteria (question 1), description of the study subjects and settings (question 2), and identification of confounding factors (question 5). The disagreement for questions 1 and 2 is largely due to the varying degree of detail with the inclusion criteria, the setting, and the independent reviewers’ interpretation of what constituted “enough” detail. For question 5, the disagreement is due to some studies not clearly identifying confounding factors. For example, some studies only use male participants, and do not identify the confounding factor of not using female participants in the study.

### 3.3. Characteristics of Included Studies 

The extracted data from included studies are provided in Table 3. A total of 24 studies are cross-sectional studies, while one study is a quasi-experimental study [41]. The countries where the studies took place varies widely, and includes; India (n = 4: [12,24,42,43]), Iran (n = 2: [7,18,19]), the United States of America (n = 2: [14,15]), Israel (n = 2: [17,44]), Nigeria (n = 2: [6,16]), the United Kingdom (n = 2: [45,46]), China (n = 2: [3,47]), Pakistan (n = 1: [7,9]), Egypt (n = 1: [5]), Slovenia (n = 1: [8]), Thailand (n = 1: [48]), Malaysia (n = 1: [49]), Korea (n = 1: [4]), Brazil (n = 1: [50]), and Canada (n = 1: [2]), with one study considered a multinational study (n = 1: [7]). Twelve studies include male drivers only [2,5,6,7,16,17,24,42,43,44,48,50], while five studies include male and female drivers [3,8,15,45,47]. Eight studies do not specify sex/gender [4,9,12,14,18,41,46,49].

### 3.4. Vehicle Type and Road Setting

The most common vehicle types are buses (n = 14: [3,4,5,6,8,12,17,41,42,43,44,45,49]), followed by trucks (n = 5: [2,9,14,48,50]), taxis (n = 4: [7,15,24,47]), or various vehicles (n = 3: [16,18,46]). The studies cater to various settings, with urban/city being the most common (n = 7: [3,12,15,17,41,42,44]), followed by highways (n = 2: [2,50]), city (n = 2: [5,8]), long-distance (n = 2: [9,16]), urban/rural (n = 1: [24]), asphalt and cobble surfaces (n = 1: [45]), intercity/intracity (n = 1: [6]), deep sea port/road (n = 1: [48]), and smooth/rough urban, suburban, rural, regional, industrial (n = 1: [49]). Six studies [4,7,18,43,46,47] do not state the setting.

### 3.5. Outcome Measures 

The most common outcome measure is the Nordic musculoskeletal questionnaire (n = 13: [3,5,6,14,15,17,18,41,42,44,47,48,49]. Other outcome measures include a self-developed or self-designed questionnaire created specifically for the study (n = 3: [2,12,50]), a numerical pain rating scale (n = 2: [4,7]), the Borg scale (n = 2: [15,48]), a Tri-axial seat pad accelerometer (n = 2: [45,46]), a self-designed questionnaire previously employed in other research (n = 2: [45,46]), the Oswestry Disability Index (n = 1: [9]), the visual analogue scale (n = 1: [43]), the Foot Function Index (n = 1: [24]), the KOSHA code H-30-2003 (n = 1: [4]), the Roland–Morris disability questionnaire (n = 1: [7]), self-administered questionnaires (n = 1: [7]), and Likert-type closed questions (n = 1: [8]).

### 3.6. Key Findings

#### 3.6.1. Musculoskeletal Disorder Sites and Severity

The most commonly reported MSK disorder site is the lower back (n = 21: [3,4,5,6,7,9,12,14,15,16,17,18,41,42,44,45,46,47,48,49,50]), followed by the neck (n = 12: [1,3,6,8,12,14,18,42,43,44,48,49]), then the shoulder (n = 11: [2,3,4,6,8,12,14,19,42,44,49]), the knee (n = 8: [3,6,12,14,18,42,44,48]), and the wrist/hand/finger (n = 7: [2,4,6,12,14,42,44]). The following sites are identified on five or fewer occasions; the upper back (n = 5: [2,6,42,44,49]), the hip/thigh/buttock (n = 4: [3,6,42,48]), the leg (n = 4: [2,4,14,46]), the ankle/foot (n = 4: [6,14,42,48]), the elbow (n = 3: [4,6,42]), the heel (n = 1: [12]), and the spine (n = 1: [8]).

Three studies present the severity of MSK disorders due to driving. One study investigates LBP severity in the last 12 months of pain, which prevented taxi drivers undertaking normal work (n = 1: [16]). One study finds that LBP intensity and rating score is the highest for truck or van drivers, ~7.5 and 6.8 out of 10 (worst possible pain), respectively (n = 1: [46]). One study reports that truck/van (29%) and taxi drivers (15%) have the greatest percentage of drivers taking more than five days off work due to LBP (n = 1: [46]). One study identifies increases in the level of disability for truck drivers as travelling hours increase (n = 1: [9]).

#### 3.6.2. Type of Vehicle and Disorder

There are four vehicle types (bus, truck, taxi, car) found across the 25 studies, and each present with varying MSK disorders; the most common sustained by drivers across the types of vehicles are lower back, neck, and shoulder. Thirteen bus/coach studies include the lower back [3,4,5,6,12,16,17,41,42,44,45,46,49], the neck (n = 9: [3,4,6,8,12,42,43,44,49]), the shoulder (n = 8: [3,4,6,12,41,42,44,49]), knee (n = 6: [3,6,12,41,42,44]), leg/ankle/feet (n = 4: [4,6,42,46]), wrist/hand/finger (n = 4: [4,6,12,42]), upper back (n = 3: [6,42,49]), hip/thigh/buttock (n = 3: [3,6,42]), arm/elbow (n = 3: [4,6,42]), heel (n = 1: [12]), and spine (n = 1: [8]) as the bodily sites of MSK disorders. Seven truck/van studies include the anatomical sites of the lower back [9,14,16,18,46,48,50], neck (n = 3: [14,18,48]), knee (n = 3: [1,14,18,48]), leg/ankle/feet (n = 3: [14,46,48]), hip/thigh (n = 1: [48]), shoulder (n = 1: [14]), and wrist/forearm (n = 1: [14]). Six taxi studies include the lower back [7,15,18,24,46,47], neck (n = 1: [18]), knee (n = 1: [18]), leg (n =1: [46]), and feet (n = 1: [24]) as the bodily sites of MSK disorders. Two car studies include the lower back [16,46] and leg (n = 1: [46]) as sites for MSK disorders.

#### 3.6.3. Factors Associated with Increased Prevalence of MSK Disorder

A variety of factors associated with increasing the risk of developing MSK disorders due to occupational driving tasks are identified. These factors range from working years and hours [2,3,5,6,7,16,47,49], to vehicle ergonomics [3,5,12,17,44,45], and whole-body vibration [3,12,45,46,49] to age [6,9,16,18], gender [3], and BMI and body weight [17,45,47].

##### Working Years and Hours

Eight studies identify the number of working years as a factor associated with the prevalence of MSK disorders amongst drivers. Of these studies, seven find that an increased number of years as a driver is a significant risk factor, with durations reported of one to five years [6], 96.38 ± 3.3 months [49], 9.1 ± 9.5 years [2], more than 10 years [5], equal to or more than 10 years [47], more than 16 years [3], and equal to or more than 20 years [16]. Arslan et al. [7] find a dose–response relationship, whereby as the number of working years increases, so does the risk of MSK disorders.

Four studies identify the number of working hours in a day to be a risk factor for MSK disorders. These studies report that 10.22 ± 3.75 h per day for truck drivers [50], more than 8 h per day for bus and taxi drivers [5,47], and 10 to 15 h per day for bus, car, and truck drivers [16] is associated with an increased risk of MSK disorders. On a weekly scale, 48.72 ± 21.50 h per week reported for truck drivers, and 36.23 ± 15.80 h per week for taxi drivers [18], are associated with an increased MSK disorder risk.

##### Vehicle Ergonomics

When considering the ergonomics of the driver’s vehicle, six studies [3,5,12,17,44,45] identify seat position/body mismatch in taxi and bus drivers as being associated with MSK disorders in drivers; six studies [3,5,12,43,44,49] identify uncomfortable steering wheel position and steering wheel tightness in bus drivers; three studies [17,44,45] identify back support position in taxi and bus drivers; while two studies [3,12] identify gear box position/control in bus drivers as being associated with MSK disorders. In bus drivers, inadequate leg space [12] and awkward postures [49] are also identified as factors associated with MSK disorders.

##### Whole Body Vibration

Five studies [3,12,45,46,49] investigate vibration doses within truck/van, taxi, and bus drivers as a risk factor for MSK disorders, with four of these studies finding significant increases in vibration as a risk factor [12,13,14,44]. Of note, these studies present vibration in a variety of ways, including three axes for vibration dose value (m/s^1.75^) [45], total vibration dose (year m^2^s^−4^) [46], relevant risk indicated with an odds ratio (OR) and 95% CI values [12,49], and self-perceived vibration dose [3] (the only study finding no significant risk associated with increases in vibration). Noting that only two studies provide comparable values, Geete et al. [12] report an OR of 2.79 (95% CI 1.50–4.70) for exposure to vibration and injury risk in bus drivers, which was higher than that reported by Tamrin et al. [49] who report an OR of 1.95 (95% CI 1.39–2.72) in relation to bus driver perceptions of exposure to vibration and risk of MSK disorders. No studies present or refer to any specific vibration thresholds.

##### Age, Sex, BMI, and Weight

Four studies [6,9,16,18] identify that older drivers are at a greater likelihood of MSK disorders compared to younger drivers. In these studies, older drivers are considered as drivers more than 45 years [16], or more than 48 years [6,18]. Rehman et al. [9] find a positive association between LBP and increasing age from 18 to 67 years. Conversely, two studies [3,17] find that younger drivers have an increased prevalence of MSK injuries as compared to older drivers. In these studies, younger drivers are considered those aged < 40 years [3] and 45 ± 9.5 years [17].

The only study that investigates the impact of sex on the likelihood of developing MSK disorder is by Szeto and Lam [3]. In their study of urban bus drivers, female drivers report a significantly higher prevalence of neck, shoulder, and knee/thigh pain, when compared to male drivers.

Increased driver BMI is identified as an associated factor for MSK disorders. BMI measures of 27.2 ± 3.9 kg/m^2^ [17] and 26.6 ± 3.8 kg/m^2^ for truck drivers, and 26.5 ± 3.9 kg/m^2^ for taxi drivers [18], and above 24 kg/m^2^ [47], are identified as being at a higher odds of MSK disorders. In terms of body weight alone, city bus drivers with LBP have a mean weight of 85.1 ± 13.3 kg when compared to a weight of 84.7 ± 19.1 kg in those without LBP [45]. However, the results are only descriptive, with no statistical analysis completed.

##### Other Associated Factors

Less frequently identified MSK disorder-associated factors are also noted. In one study [15] of taxi drivers, LBP is more likely to occur with higher perceived physical exertion while driving, 4.10 ± 2.00 au, compared to those with less perceived physical exertion while driving, 3.00 ± 2.1 au, measured on a Borg CR10 scale. Also in research on taxi drivers [47], LBP is more likely to occur in drivers working night shifts (9 pm to 6 am) than those working day shifts (6 am to 9 pm).

A study of bus drivers [3] finds MSK disorders or discomfort are more likely to occur in drivers who have less than five years work experience when compared to drivers working more than six years, measured using a Nordic musculoskeletal questionnaire. In another study of bus drivers [17], there are more complaints of limited rest periods during a working day for those with LBP (39.3%) when compared to those without LBP (28.1%). Bus drivers with weak supervision of working conditions (such as setting the position of rear view mirrors and steering wheel), excessive workloads (such as long working hours), needing more rest and recovery of strength (such as active rest and day rest), and poor to moderate general health perceptions (such as alcohol consumption, smoking habits, and poor eating habits) are also found to be at an increased risk of LBP [8]. Sangiamsak and Thetkathuek [48] conducted a study in truck drivers, and find that MSK symptoms and perceived discomfort are more likely to occur with increased driving distance, when compared to those drivers who travel short distances.

#### 3.6.4. Associated Factors-Decreasing Risk or MSK Disorders

Four studies investigate various methods of minimizing MSK disorders that are a consequence of occupational driving [4,17,41,47]. One study [41] investigates the effects of knowledge and education of pain and risk factors in truck drivers through a Health Belief Model, which consisted of three educational sessions. The sessions consisted of PowerPoint presentations, educational pamphlets, introduction to health professionals, meeting drivers with MSK disorders, and undertaking supervised physiotherapy covering exercise techniques and familiarization with all stages of physical activity. This intervention improves self-efficacy, the perception and awareness of MSK disorder risks, and the consequences of prolonged inactivity [41].

In a study by Lee and Gak [4], the effects of four weeks of self-stretching on bus drivers is investigated. The intervention consisted of self-stretching exercises, self-checklist documentation, and education by physical therapists about stretching methods and precautions. The researchers conclude that the stretching intervention produces a significant decrease in pain and MSK symptoms in the neck and shoulders (6.17 ± 1.51 pre-intervention, and 3.21 ± 1.87 post-intervention), as measured by a numeric rating scale. Overall, the intervention finds stretching can decrease MSK pain and symptoms, and improve flexibility in occupational drivers [4].

Wang et al. [47] investigate LBP among taxi drivers using a three-stage questionnaire. It is found that more than one rest day a month, eight or more hours of sleep, and those who report any physical activity report a decreased likelihood of LBP over a 12 month period. This investigation finds that more rest days, longer sleep duration, and more physical activity decreases rates of reported LBP in taxi drivers.

One study [17] investigates ergonomic, leisure time physical activity, and occupational–psychosocial risk factors with bus drivers using a two-part questionnaire. This study finds that regular physical activity, such as walking or sports activities, is reported more frequently in the non-LBP group (67.3%) when compared to the LBP group (48.5%).

## 4. Discussion

The aim of this systematic review was to collect, critically appraise, and synthesize the findings of research investigating WRMSDs associated with occupational driving, and concomitant contributing factors to these injuries, in order to develop a volume of evidence to inform mitigation strategies. Representing 14 countries and 5 road vehicle types, the 25 studies informing the review score, on average, 69% when critically appraised. The included studies are, on average, deemed to be of ‘good’ methodological quality. LBP is the most represented MSK condition among occupational drivers, whether they be operating a bus, coach, truck, van, or taxi. However, injuries to various other MSK sites, including the neck, shoulder, knee, and feet, are also reported. Few studies provide insight into risk mitigation strategies. Ultimately, driver education regarding pain and risk factors, stretching, and addressing lifestyle factors, such as rest, more sleep, and physical activity, are considered to reduce the risk of MSK disorders associated with occupational driving.

### 4.1. Lower Back Pain

A total of 22 out of 25 studies [2,3,4,5,6,7,9,12,14,15,16,17,18,19,42,44,45,46,47,48,49,50] (88%), identify LBP as being a common MSK disorder for occupational drivers. With 84% of occupational drivers expected to experience LBP in their lifetime [1], truck/van [46], bus [12], and taxi drivers [46] are found to be the most affected when compared to other road-specific, occupational driving vehicles. Furthermore, truck/van (29%) and taxi drivers (15%) are reported as having the greatest percentage of drivers taking more than five days off work due to LBP [46]. Given this common MSK disorder across the studies, it is not surprising that frequently identified risk factors for occupational MSK disorders in drivers include long hours driving in a sustained position, number of years undertaking occupational driving, vehicle ergonomics, and whole-body vibration.

Data reported in this review suggest that shift duration of more than 8 h [5,47], and weekly driving hours of 36 to 48 h [18], account for an increased risk of LBP in occupational drivers. In addition, approximately one third of the studies find that the number of years in a driving occupation contributes to LBP, and the risk increases with number of working years [6,7,16]. While occupational sitting itself may not be a risk factor for LBP [52], remaining in a prolonged sedentary position for many hours, with minimal or short breaks, can lead to muscular fatigue, which strains the lumbar region [47]. A fixed and/or awkward sitting posture produces and exacerbates a negative chain of events. This begins with continual force or overloading the joints [18], which leads to gradual, repeated trauma [16] that affects connective and soft tissue, and results in pain [6]. Furthermore, extended sitting while driving decreases mobility, coordination, and motion control [49], causes poor circulation, and static muscle contractions, as well as degenerative change or impairment [2], such as annular tears and disc herniation [49]. 

The impact of spending long durations in a sustained position is further exacerbated by poor vehicle ergonomics, which is another mechanism contributing to LBP. For example, the driver’s seat comfort and design can cause further injury risk to occupational drivers [4,5,6,7,8,9]. Factors including seat cushioning, leg space, and seat position/body mismatch can contribute to driver comfort, or lack of, which may cause drivers to adopt and remain in awkward and uncomfortable body postures, such as slump sitting or uneven weight distribution, for an extensive period [5,44]. As previously discussed, this can place mechanical stress on the spine and surrounding soft tissue structures, causing increased pressure on intervertebral disc, which may ultimately lead to LBP or other MSK disorders [12]. Additionally, having seats with inadequate back support for drivers can contribute to increased risk, by adding further compressive load on the lower back, or requiring extra demands on active and supporting muscles, causing irregular and unhealthy postures, such as inducing forward head lean and protracted shoulders [49]. Thus, while poor vehicle ergonomics may not be a source of LBP risk itself, it may exacerbate the risk associated with long periods of sustained sitting.

Finally, whole-body vibration as a mechanism contributing to LBP in occupational drivers is frequently identified in studies informing this review [3,12,45,46,49]. This is, again, not surprising, given that vehicle transit is consider the most common and the most harmful means of inducing whole-body vibration [53], and that while prolonged sitting induces greater biomechanical loading on the lumbar intervertebral discs [54], the continuous low-load vibrations can exacerbate “creep” in the soft tissues [53]. As such, when whole-body vibration is combined with sitting for prolonged periods [12] and poor seated posture [45,46], it is understandable that driving shifts of 6 to 8 h [49] are noted as a risk for increasing LBP. Apart from direct vibrational impacts on the MSK system, whole-body vibration is also noted as increasing the risk of muscular fatigue [49], with fatigue, in turn, known to increase the risk of driver MSK LBP [47]. Furthermore, whole-body vibration may negatively impact the performance of tasks immediately following exposure [53]. As such, exposure to whole-body vibration during occupational driving may lead to MSK disorders when the driver exits the vehicle and must perform a physically demanding task (such as unloading their van/truck). This potential second-order effect of vibration bears consideration, given that a leading mechanism of MSK disorders (34–40% of injuries) in delivery truck drivers is overexertion from lifting and lowering packages and boxes [55]. Finally, and of most concern, the study by Okunribido et al. [45] finds that vibration dose values for all three bus types (mini-bus, double decker, and single decker buses) exceeds the European safe daily exposure limit of 9.1 m/s^1.75^ [56]. Furthermore, all buses breach the maximum threshold, in which a worker is not to be exposed, under any circumstances, to 21 m/s^1.75^ when driving on cobble surfaces [56]. This finding may help explain why truck/van drivers, are found to report the highest LBP intensity (~7.5 and 6.8 out of 10, respectively) [46], and highlights the requirement for controls to be put in place to reduce driver exposure to vibrations at these thresholds [56].

### 4.2. Other Musculoskeletal Disorders 

LBP is the most common and frequently identified MSK disorder in occupational drivers, while others include regions of the neck [1,3,6,8,12,14,18,42,43,44,48,49]), the shoulder [2,3,4,6,8,12,14,19,42,44,49]), the knee [3,6,12,14,18,42,44,48], and the foot [24]. These sites are supported by findings in other studies of occupational drivers [11,20,21,22,23]. It is possible that extensive periods of time in awkward postures contributes to mechanical stress and compensation on various joints, structures, and muscles in the neck, leading to dysfunction and/or pain. The role of occupational drivers requires prolonged sitting with muscles surrounding the neck, that support the head, becoming fatigued, causing drivers to adopt a forward head lean posture, which may cause neck pain [43,57]. A similar cause of neck pain is noted in office workers, due to prolonged computer use [58]. Additionally, as previously mentioned, drivers, similar to the aforementioned computer officer workers, may adopt abnormal postures due to uncomfortable seating and lack of support, such as leaning to one side or slumping, which can create further problems in MSK pain and dysfunction, leading to pains in the neck, spine, and upper back [3,4,6,8,12,42,43,44,49,58].

Shoulder disorders are also commonly reported workplace issues in occupational drivers [2,3,4,6,8,12,14,19,42,44,49]. There are many mechanisms for shoulder disorders, such as steering wheel tightness [3,44], awkward seated postures [44], incorrect or poor seat dimensions, which result in greater shoulder stiffness [2], and seats without upper back support [12]. All of these factors increase the load on the cervical spine and upper back, and can lead to shoulder pain. As with LBP, increased exposures to these factors increases the risk of MSK disorders, with a higher number of working years identified as an increased risk factor for shoulder pain associated with occupational driving [3].

### 4.3. Other Considerations

Further considerations that are identified through the studies are the demographics of the participants, including age and BMI. Interestingly, six studies [3,6,9,16,17,18] found age to be a risk factor. Two studies [3,17] define younger drivers (<40 years old) as being at greater risk of MSK disorders; conversely four studies [6,9,16,18] determine that older adults (>45 years old) are at a greater risk of MSK disorders. While age as a potential risk factor cannot be clearly established, the two studies [3,17] that found younger drivers at a higher risk than older drivers, focus on bus drivers, while the remaining studies focus on other road vehicles. Furthermore, in their study, Szeto and Lam [3] consider younger drivers to be at a greater risk due to less job experience. This finding is supported in the work by Lockie et al. [59], who find that older police officers are less likely to have a vehicle accident when compared to younger officers. As such, a trade-off may exist between driving experience and occupational exposure (e.g., whole body vibration). 

Four studies [17,18,45,47] discuss the impact of BMI on injury risk, and find associations between higher BMI and an increased risk of MSK disorders among occupational drivers. However, noting that higher BMI is, in general, associated with an increased risk of LBP, whether these disorders are due to occupational driving specifically cannot be assumed [60], as both BMI and prolonged sitting are associated with LBP in other occupations, from teaching [61] to textile work [62].

### 4.4. Risk Reduction

The identified MSK disorders and common occupational driving associated mechanisms can have serious implications on the health of occupational drivers, affecting the longevity and quality of their physical well-being, livelihood, earning capacity, and mental state [4,12]. More broadly, these disorders can incur financial costs for companies, health systems, society, and individuals, as well as increase absenteeism, compensation and claims costs, and work-related errors and stress [63]. As such, it is imperative that measures are taken to mitigate MSK disorder risk for occupational drivers. In order to prevent, or help reduce, the possibility of shoulder pain in occupational drivers, it is found that a four week intervention of stretching reduced shoulder pain by 28% [4]. This finding is supported by the wider literature. For example, a randomized control trial of a four week neck and shoulder stretching intervention for officers workers with neck pain reports significant improvements (*p* < 0.05) in Northwick Park neck pain questionnaire scores following the intervention [64].

Another intervention, which involved using educational material, meeting health professionals and drivers with MSK disorders, and exercise and physical activity, decreases the frequency and distribution of pain decreases in the shoulder by 26%, lower back by 17%, and the knee by 13% after the intervention [41]. This finding is consistent with the systematic review by Steffens et al. [65]. In the review, it was noted that while there is moderate- to very low–quality evidence that education alone has an effect on LBP, when education is combined with exercise, as per the study by Ghasemi and Pirzadeh [41], there is moderate-quality evidence suggesting reductions in the risk of LBP. 

Finally, more physical activity, more rest, and longer sleep duration are found to reduce the risk of MSK disorders, notably LBP, in occupational drivers [47]. Again, these findings are supported by the wider literature. For example, the amount and quality of sleep are known risk factors in occupational settings, with the lack of sleep considered to impact on both safety compliance and safety participation, in turn, increasing the risk for workplace injuries [66], and musculoskeletal pain in general [67]. Overall, physical activity [68], reduced rest [69], and poor sleep [70] increase workplace fatigue, which constitutes a risk to workplace safety. Furthermore, in drivers specifically, lack of sleep increases drowsiness and the risk of falling asleep while driving, thereby increasing the risk of vehicle accidents [71]. As such, strategies to increase physical activity, rest (i.e., more breaks), and sleep quantity may be of benefit to occupational drivers.

While not the intent of this review, considerations of these risk-mitigating factors may help inform means to reduce MSK disorders in occupational drivers. However, of note, when considered against the hierarchy of controls for risk mitigation, the findings presented above represent the least effective controls (personal protective controls) [72], and generally place the responsibility of risk mitigation on the individual driver. As such, future studies should look to controls higher up the hierarchical tree. For example, means of eliminating (e.g., eliminating vibration exposure), or substituting (e.g., substituting elements causing vibration with other lower/no vibration controls, or substituting different seating), risks warrant further investigation. Both of these aforementioned hierarchical controls can be informed by engineering controls (e.g., vibration dampening devices or changes to seat/vehicle ergonomics). Finally, administration controls (mandatory rest periods, length of driving shifts, driving routes, etc.) may, likewise, be of benefit. In addition, possible considerations into whether physiotherapy, general health, well-being, and educational interventions should be made mandatory in occupational driving workplaces and, thus, an integral part of transport industry business structure, bears consideration. 

### 4.5. Limitations and Future Research

There are three potential limitations associated with this review. Firstly, while the broad geographical representation of the studies is a strength of this review, it is also a limitation, given that different countries present differences in road regulations, and levels of surveillance and enforcement of safety standards. Secondly, while the different types of driving settings, such as rural [24], regional [14], city areas [8], and highways [50] are identified in several studies, details as to the condition of these roads (e.g., bitumen, gravel, wet roads, affected by snow) is limited. Operating on such unstable surfaces, and in potentially dangerous conditions, could increase muscle tension throughout the upper and lower limbs due to increased grip and tension, which could exacerbate the risk of MSK disorders and impact the results. Finally, despite a comprehensive search of literature, only five [3,8,15,45,47] studies identify female drivers as participants in their cohorts, with the majority of studies focusing on male populations. The one study that did discuss gender differences finds that females have a high rate of MSK disorders when compared with their male counterparts [3]. It can be hypothesized that the reason for this is due to occupational driving being more male dominant, and as such, ergonomic designs (such as the seat), being more suited to males; this supposition is supported by research in body armor design [73]. However, future research is required to support both this supposition and the findings of increased MSK rates in female drivers. Furthermore, based on the findings of the methodological ratings of the included reviews, future studies should explore and report and, if possible, adjust for confounding factors associated with MSK disorders in occupational drivers. 

## 5. Conclusions

A wide and diverse range of studies, spanning a multitude of countries and road vehicle types, were found investigating workplace MSK disorders in occupational drivers. This review adds an up-to-date profile of musculoskeletal disorders associated with occupational driving, of note given the potential changes to vehicle designs in the last 15 years, in order to inform mitigation strategies. LBP, in particular, is found to be frequently reported as an MSK disorder, although other bodily sites include the neck, shoulders, upper back, and knees. Common risk factors for these occupations driving disorders include long hours in a prolonged sitting position and years in an occupational driving profession, vehicle ergonomics, and whole-body vibration. Addressing these risk factors, through stretching programs, being more physically active in general, together with driver education, may help mitigate these risks. However, further research is needed to investigate effective intervention strategies to reduce occupational MSK injuries and disorders in occupational drivers, based on the reported bodily sites of injury and associated risk factors. In addition, these investigations should be derived from the hierarchy of controls, so as to provide for the most effective control strategies to mitigate risks. 

## Figures and Tables

**Table 1:** Database and relevant search terms.

Database	Search Terms	Filters
PubMED	((driver[tiab] OR long-haul[tiab] OR long-haul[tiab] OR drivers[tiab] OR driving[tiab]) AND (MSD[all] OR “Back Pain”[all] OR Whiplash[all] OR Spine[all] OR Hip[all] OR Knee[all] OR Pain[all] OR Injury[all] OR injuries[all] OR injured[all] OR “Musculoskeletal Pain”[Mesh]))	Human, English, year (2006–2021)
EBSCO HOST/CINAHL	(((TI driver OR AB driver) OR (TI long-haul OR AB long-haul) OR (TI long-haul OR AB long-haul) OR (TI drivers OR AB drivers) OR (TI driving OR AB driving)) AND (MSD OR “Back Pain” OR Whiplash OR Spine OR Hip OR Knee OR Pain OR Injury OR injuries OR injured OR (MH “Musculoskeletal Pain”+)))	Human, year (2006–2021)
SPORTDiscus	(((TI “driver” OR AB “driver”) OR (TI “long-haul” OR AB “long-haul”) OR (TI “long-haul” OR AB “long-haul”) OR (TI “drivers” OR AB “drivers”) OR (TI “driving” OR AB “driving”)) AND (TX “MSD” OR TX “Back Pain” OR TX “Whiplash” OR TX “Spine” OR TX “Hip” OR TX “Knee” OR TX “Pain” OR TX “Injury” OR TX “injuries” OR TX “injured” OR DE “Musculoskeletal Pain”))	English, year (2006–2021)
Web of Science	((driver OR long-haul OR long-haul OR drivers OR driving) AND (MSD OR “Back Pain” OR Whiplash OR Spine OR Hip OR Knee OR Pain OR Injury OR injuries OR injured OR “Musculoskeletal Pain”))	English, year (2006–2021) Additional filters (e.g., exclude surgery, neurology, oncology etc.)

**Table 2:** Eligibility criteria.

Inclusion Criteria	Exclusion Criteria
The target population of the included research must be drivers (occupational, professional, or commercial) and include truck, taxi, bus, or long-haul drivers. For this study drivers who operate trains, trams, or other non-road vehicles are not included; The injury needed to occur as a result of operating or driving a motor vehicle; Over 16 years of age (common legal driving age); Published since 2006 (last 15 years) due to changes in vehicle design [34,35,36]; and Studies must be peer-reviewed and original research.	Injuries not sustained while driving; Accident/crash-related injuries; Injuries not clearly defined; Vehicles other than road vehicles included in the data Pre-existing injuries; or Altered driving surface (dirt racing track)

**Table 3:** Data extraction table.

Author/Date	Participants	Vehicle/Setting	Outcome Measure	Key Findings				CAS
Ahire and Shukla, 2021 [24]	♂ drivers with 10 years driving experience and a pain complaint (n = 90) Age range: 40–50 years	Taxi/urban and rural	FFI	FFI score left foot: 64% FFI score right foot: 91%				50%
Alperovitch-Najenson et al., 2010 [44]	♂ drivers with and without MSK complaints Total drivers (n = 359) Mean age: 46.0 ± 9.8 years Mean BMI: 27.0 ± 3.9 kg/m^2^ Mean work experience: 18.0 ± 11 years Drivers with neck pain (n = 76) Mean age: 45.2 ± 9.8 years Mean BMI: 27.0 ± 4.1 kg/m^2^ Mean work experience: 17.7 ± 10.5 years Drivers without neck pain (n = 283) Mean age: 46.3 ± 9.8 years Mean BMI: 27.0 ± 3.8 kg/m^2^ Mean work experience: 18.2 ± 11.1 years	Bus/urban/city	NMQ	Lower back, neck, shoulder, knee most common MSK pain: Lower back: 45% (n = 162) Neck: 21% (n = 75) Shoulder: 15% (n = 54) Knee: 10% (n = 36) Upper back: 8% (n = 29)				75%
				Prevalence of MSK symptoms		Drivers with neck pain (n = 76)	Drivers without neck pain (n = 283)	
				Shoulder: Elbow: Wrist: Upper back:		41% (n = 31) 7% (n = 5) 9% (n = 7) 12% (n = 17)	8% (n = 22) 2% (n = 6) 1% (n = 4) 5% (n = 13)	
				MSK symptoms in drivers with neck pain (reference: no neck pain): Shoulder: OR 8.10 (95% CI 4.30–15.30), *p* < 0.001 Elbow: OR 3.20 (95% CI 0.90–10.90), *p* < 0.05 Wrist: OR 7.00 (95% CI 2.00–21.80), *p* < 0.001 Upper back: OR 5.90 (95% CI 2.70–12.90), *p* < 0.00				
				Drivers with neck pain more frequently complained of: − uncomfortable seat: OR 2.20 (95% CI 1.20–4.30), *p* < 0.05; − uncomfortable back support: OR 2.30 (95% CI 1.20–4.20), *p* < 0.01; − uncomfortable steering wheel: OR 2.20 (95% CI 1.10–4.50), *p* < 0.05.				
Alperovitch-Najenson, Santo et al., 2010 [17]	♂ drivers (n = 361) With LBP (n = 164) Mean age: 45.0 ± 9.5 years Mean BMI: 27.2 ± 3.9 kg/m^2^ Mean weight: 27.2 ± 3.9 kg Mean height: 1.75 ± 0.07 m Without LBP (n = 197) Mean age: 47.0 ± 10.0 years Mean BMI: 26.8 ± 3.8 kg/m^2^ Mean weight: 26.8 ± 3.8 kg Mean height: 1.74 ± 0.07 m	Bus/urban/city	NLBPQ	LBP complaint: 45% (n = 164) No LBP: 55% (n = 197) Drivers in both groups are overweight, but no significant difference between groups (*p* = 0.35) With LBP: 27.2 ± 3.9 kg/m^2^ Without LBP: 26.8 ± 3.8 kg/m^2^ Drivers in LBP group significantly younger *p* < 0.05: With LBP: 45.0 ± 9.5 years Without LBP: 47.0 ± 10.0 years Drivers with LBP more frequently complained of: Uncomfortable seat: OR 2.60 (95% CI 1.40–5.00), *p* ≤ 0.01 Uncomfortable back support: OR 2.50 (95% CI 1.40–4.50), *p* ≤ 0.01 Inadequate rest period during the working day OR 1.60 (95% CI 1.00–2.60), *p* ≤ 0.05. Drivers who participated in regular physical activities are significantly (*p* < 0.01) fewer in the LBP (49%) versus the non-LBP (67%) group				75%
Aminian et al., 2016 [18]	Drivers with at least one year professional driving, a negative history of surgery related to MSK disorders, and a negative history of trauma leading to severe injury or fracture in lumbar, neck, elbow, arm (n = 734) Truck drivers (n = 366) Mean age: 39.8 ± 9.3 years Mean BMI: 26.6 ± 3.8 kg/m^2^ Mean work hours: 48.7 ± 21.5 (weekly) Mean work experience: 13.2 ± 9.0 (unit of measure not provided) Taxi drivers (n = 368) Mean age: 41.9 ± 9.9 years Mean BMI: 26.5 ± 3.9 kg/m^2^ Mean work hours: 36.2 ± 15.8 (weekly) Mean work experience: 15.7 ± 9.8 (unit of measure not provided)	Truck, taxi, not stated	NMQ	Association of driving a truck (reference: taxi drivers) with: Neck pain: OR 2.96 (95% CI 1.23–7.13) Knee pain: OR 4.95 (95% CI 1.81–13.54) MSD: OR 1.63 (95% CI 1.08–2.44) LBP: OR 2.35 (95% CI 1.12–4.93) Being heavier (OR 1.40 (95% CI 1.03–1.91))/shorter (OR 0.70) increased knee pain risk Increased work hours per week increased neck pain risk OR 1.02 (95% CI 1.00–1.03) *p* = 0.049 Age LBP increased with older age OR 1.07 (95% CI 1.02–1.12) Older age effective risk factor for MSD OR 1.05 (95% CI 1.02–1.09) Neck pain: OR = 4.64 (95% CI = 2.29–9.40) Truck drivers: 12% (n = 42) Taxi drivers: 3% (n = 10) Knee pain: OR 5.28 (95% CI 2.31–12.08) Truck drivers: 9% (n = 34) Taxi drivers: 2% (n = 7) Disorder in one or two knees in truck drivers is 5.5 (95% CI 2.3–12.1) times more than taxi drivers LBP most common symptom in both groups: Truck: 20% Taxi: 14%				75%
Andrusaitis et al., 2006 [50]	♂drivers with more than one year of professional driving Total driver (n = 410) Mean age: 40.2 ± 10.2 years Mean BMI: 26.8 ± 3.8 kg/m^2^ Mean height: 1.72 ± 0.06 m Mean work experience: 15.6 ± 9.9 years Mean work hours: 9.9 ± 3.6 (daily) Drivers with LBP (n = 242) Mean age: 39.8 ± 10.0 years Mean BMI: 27.0 ± 3.8 kg/m^2^ Mean height: 1.72 ± 0.07 m Mean work experience: 15.3 ± 9.5 years Mean work hours: 10.2 ± 3.8 (daily) Drivers without LBP (n = 168) Mean age: 40.7 ± 10.5 years Mean BMI: 26.5 ± 3.7 kg/m^2^ Mean height: 1.71 ± 0.06 m Mean work experience: 15.9 ± 10.5 years Mean work hours: 9.4 ± 3.2 (daily)	Truck/highways	LBP questionnaire developed for study	LBP: 59% (n = 242) No LBP: 41% (n = 168) Of the 242 with LBP: Occasional: 31% Constant: 18% Sometime in professional lives: 10% Number of working hours associated with LBP: OR 1.07 (95% CI 1.01–1.13)				75%
Arslan et al., 2019 [7]	♂ drivers with one year of professional driving experience (n = 1200) Pakistan Mean age: 42.8 ± 9.3 (20–60) years Mean weight: 80.4 ± 10.8 kg Mean BMI: 27.3 ± 3.42 kg/m^2^ Iran Mean age: 47.4 ± 8.9 (20–60) years Mean weight: 81.9 ± 10.7 kg Mean BMI: 27.7 ± 3.4 kg/m^2^	Taxi/not stated	Self-administered questionnaire NPRS RMDQ	Prevalence of LBP Point: One week: One year: Lifetime:		Pakistan: 27% 36% 50% 78%	Iran: 37% 43% 54% 72%	87.5%
				NPRS		4.15 ± 1.42	4.00 ± 1.57	
				RMDQ		7.76 ± 2.50	7.71 ± 2.99	
Burgel and Elshatarat, 2017 [15]	♂/♀ drivers who drive a minimum of 20 h per week (n = 129), ♂ (n = 121), ♁ (n = 8) Mean age: 45.3 ± 10.8 years Mean BMI: 27.4 ± 4.8 kg/m^2^ Mean driving experience: 9.8 ± 8.4 years Mean work hours: 40.9 ± 13.0 h (weekly)	Taxi/ urban, city	NMQ Borg CR10 scale	LBP: 63% (n = 81)/No LBP: 37% (n = 48) Of the 63%, 35% (n = 28) report LBP prevented them from doing normal work in last 12 months 61% (n = 49) report trouble from LBP in last 7 days Drivers who have higher physical exertion (4.10 ± 2.00) more likely to report 12 month LBP, compared to those who have less physical exertion (3.00 ± 2.10 *p* = 0.003)				87.5%
Geete et al., 2013 [12]	Drivers (n = 60) Age range: 35–50 years	Bus/urban, city	Self-designed questionnaire	Prevalence of MSK pain: 80% Distribution of MSK pain: Low back: 70% Neck: 55% Shoulder: 48% Knee: 31% Wrist: 23% Heel: 20% Association of back pain with workstation risk factors: Uncomfortable seat position: OR 3.15 (95% CI 1.60–5.20), *p* < 0.05 Steering wheel position: OR 2.00 (95% CI 1.40–4.40), *p* < 0.01 Inadequate leg space: OR 2.05 (95% CI 1.40–4.50), *p* < 0.01 Vibration: OR 2.79 (95% CI 1.50–4.70), *p* < 0.05 Association of neck pain with workstation risk factors: Uncomfortable seat position: OR 2.70 (95% CI 1.50–4.90), *p* < 0.01 Uncomfortable gear position: OR 1.80 (95% CI 1.20–2.70), *p* < 0.05 Steering wheel position: OR 2.00 (95% CI 1.20–4.50), *p* < 0.05 Vibration: OR 2.50 (95% CI 1.50–4.90), *p* < 0.01 Association of shoulder pain with workstation risk factors: Uncomfortable seat position: OR 2.50 (95% CI 1.50–4.80), *p* < 0.01 Uncomfortable gear position: OR 1.75 (95% CI 1.10–3.70), *p* < 0.05 Steering wheel position: OR 2.00 (95% CI 1.10–4.50), *p* < 0.01 Association of knee pain with workstation risk factors: Uncomfortable seat position: OR 1.50 (95% CI 1.00–2.40), *p* < 0.05 Inadequate leg space: OR 1.85 (95% CI 1.20–2.70), *p* < 0.01 Association of wrist pain with workstation risk factors: Uncomfortable seat position: OR 1.50 (95% CI 1.10–2.10), *p* < 0.05 Steering wheel position: OR 1.90 (95% CI 1.20–3.10), *p* < 0.05 Vibration: OR 1.90 (95% CI 1.40–3.50), *p* < 0.01 Association of heel pain with workstation risk factors: Uncomfortable gear position: OR 2.10 (95% CI 1.40–3.20), *p* < 0.01 Vibration: OR 1.57 (95% CI 1.00–2.40), *p* < 0.05				37.5%
Ghasemi and Pirzadeh, 2021 [41]	Full time drivers with at least one year driving experience, no health conditions, and no history of lower back or knee surgery, or relevant health problems (n = 60) Age range: 32–56 years	Bus/urban, city	NMQ	Frequency distribution of pain		Before intervention:	After intervention:	67.5%
				Shoulder: Low back: Knee:		43% 37% 23%	17% 20% 10%	
Hakim and Mohsen, 2017 [5]	♂ drivers with six months of field work (n = 180) Mean age: 37.5 ± 9.2 years	Bus	NMQ	LBP significantly higher for those worked > 10 years OR 2.42 (95% CI 1.23–4.87) LBP 6.6 times higher for those worked > 10 years than those worked 10 years or less OR 6.64 (95% CI 1.35–32.8), *p* = 0.020 Those worked > 8 h/day have higher frequency of LBP (84%) OR 2.93 (95% CI 1.45–5.93) Uncomfortable seat higher significant LBP (82%) OR 2.83 (95% CI 1.43–5.59) Uncomfortable steering wheel higher significant LBP (82%) OR 2.08 (95% CI 1.01–4.31)				37.5%
Kim et al., 2016 [51]	Drivers with one year employment with the current company, and a regular work shift of 6 to 15 h (n = 96) Mean age: 48.2 ± 7.6 years Mean weight: 104.9 ± 25.5 kg Mean BMI: 32.3 ± 6.9 kg/m^2^ Mean height: 179.4 ± 7.7 cm Mean driving experience: 18.9 ± 10.7 years	Truck (regional or long-haul)/ regional	10-point pain scale adopted from NMQ	Despite 96 participants in study, only 69 completed 10-point scale. Region: prevalence% (N). Low back: 73% (n = 50); pain score: 2.90 ± 2.00 (95% CI 2.40–3.40) Shoulder: 55% (n = 38); pain score: 2.90 ± 2.50 (95% CI 2.3–3.40) Neck: 51% (n = 35); pain score: 2.70 ± 2.40 (95% CI 2.10–3.30) Knee: 42% (n = 29); pain score: 2.40 ± 2.50 (95% CI 1.80–3.00) Wrist/forearm: 36% (n = 25); pain score: 2.20 ± 2.60 (95% CI 1.60–2.80) Ankle/feet: 32% (n = 22); pain score: 1.70 ± 2.30 (95% CI 1.20–2.20) Leg pain/sciatic: 26% (n = 18); pain score: 1.40 ± 2.20 (95% CI 0.90–1.90)				75%
Kresal et al., 2015 [8]	♂/♁ drivers (n = 145) ♂: 144 ♁: 1 <30 years (n = 17) 31–40 years (n = 32) 41–50 years (n = 37) >51 years (n = 59)	Bus/city	Likert-type close-ended questions	Total of 74%: neck pain, spine pain, headaches associated with jobs (n = 107) Total of 12%: no connection between job/health conditions (n = 17) Total of 15%: job/health condition connection exists time to time (n = 21) Risk factors statistically significant for LBP: Weak supervision of working conditions, *p* = 0.040; Excessive workload, *p* = 0.012; Need for rest and recovery of strength, *p* = 0.006; Moderate/poor general health perception, *p* = 0.001.				75%
Lalit et al., 2015 [42]	♂ drivers with at least one year driving experience who spend a minimum of four hours a day in sitting position (n = 300) Mean age: 42.6 ± 5.7 (25–50) years Mean weight: 75.8 ± 12.1 kg Mean height: 171.81 ± 6.06 cm Mean work experience: 19.8 ± 6.6 years	Bus/urban, city	NMQ	Prevalence of WRMSDs: 53% (n = 159) LBP: 30% Neck pain: 17% Knee pain: 15% Shoulder: 6% Ankle/feet: 6% Upper back: 4% Hip/thigh: 4% Elbow: 1% Wrist/hand: 1%				67.5%
Lee and Gak, 2014 [4]	Drivers (n = 81) (Note: error in published Table 1, Table 2 and Table 3, which states n = 80) Mean age: 49.4 ± 8.2 years Mean weight: 70.6 ± 8.0 kg Mean height: 170.8 ± 4.89 cm Mean work experience: 10.2 ± 7.8 years	Bus/not stated	Symptom research form KOSHA code H-30-2003 NPRS	MSK symptoms: (n = 81) Neck: 34% (n = 27) Shoulder: 42% (n = 34) Arm/elbow: 6% (n = 5) Hand/wrist/finger: 6% (n = 5) Lumbar: 34% (n = 27)Leg/foot: 19% (n = 15) Significant decrease in pain after bus drivers completed self-stretching intervention, *p* < 0.05 Pre-test: 6.17 ± 1.51 Post-test: 3.21 ± 1.87 A significant decrease in MSK symptoms in neck/shoulder (*p* < 0.05) after self-stretching intervention: Neck pre-test: 34% (n = 27); neck post-test: 25% (n = 20) Shoulder pre-test: 42% (n = 34); shoulder post-test: 35% (n = 28)				37.5%
Maduagwu et al., 2021 [6]	♂ drivers with at least one year driving experience, and no traumatic road or work accidents (n = 250) Mean age: 32.14 ± 10.67 (18–66) years Work experience 1–5 years: 45 ± 18.0 Work experience 6–10 years: 76.0 ± 30.4 Work experience ≥ 11 years: 129 ± 51.6	Bus (commercial minibus)/intra/inter-city routes	NMQ	>48 years have highest prevalence of MSK (WMSDs): 43% (n = 20) Those with 1–5 years working experience have highest prevalence of MSK (WMSDs): 29% (n = 13) A total of 53 participants have WRMSDs, giving a 12 month prevalence of 21%				100%
						12 month prevalence	Weekly prevalence	
				Lower back		72%	36%	
				Neck:		40%	17%	
				Shoulder:		53%	23%	
				Elbow:		25%	8%	
				Wrist/hand:		34%	15%	
				Upper back:		38%	11%	
				Hip/thigh/buttock:		40%	11%	
				Knee:		45%	19%	
				Ankle/foot:		36%	36%	
Okunribido et al., 2008 [46]	Drivers with at least one year of driving experience; at least five years driving experience in their current or immediate past job There are 453 drivers in total, but 213 are extracted from truck/van, taxi, bus, police as these groups are relevant to our data. Police (n = 58); Mean age: 34.5 ± 5.9 years Mean weight: 83.1 ± 11.4 kg Mean BMI: 26.0 ± 2.7 kg/m^2^ Mean height: 178.6 ± 6.74 cm Truck/van (n = 64) Mean age: 46.9 ± 11.0 years Mean weight: 85.9 ± 14.8 kg Mean BMI: 27.7 ± 4.5 kg/m^2^ Mean height: 176.4 ± 6.67 cm Bus (n = 61) Mean age: 47.6 ± 10.4 years Mean weight: 84.9 ± 15.7 kg Mean BMI: 28.3 ± 4.4 kg/m^2^ Mean height: 172.9 ± 8.45 cm Taxi (n = 30) Mean age: 49.3 ± 8.3 years Mean weight: 88.5 ± 18.5 kg Mean BMI: 28.3 ± 4.7 kg/m^2^ Mean height: 176.4 ± 7.40 cm Control (n = 49) Mean age: 40.00 ± 8.38 years Mean weight: 79.70 ± 14.09 kg Mean BMI: 25.90 ± 3.62 kg/m^2^ Mean height: 175.2 ± 7.86 cm	Bus, truck/van taxi, car (police car), not stated	Self-designed questionnaire previously employed in other research Tri-axial seat pad accelerometer (whole- body vibration meter 2.0) Posture scores	Previous 12 month LBP prevalence: Truck/van, taxi drivers often experience LBP and >50% reported LBP pain intensity (~7.5) is highest for truck/van drivers Suffering > 6 episodes of LBP: truck/van (68%) and taxi (68%) Most truck/van, taxi drivers report pain lasting ≥ three days, and need to take 2 or more months off work				75%
				Previous 7 day LBP prevalence: Taxi driver associated with highest prevalence (44%) Truck/van drivers have highest rating score for LBP intensity (6.8) Taxi (62%) have highest percentages of suffering >4 episodes of LBP Most truck/van (29%) and taxi drivers (15%) have greatest percentage of taking >5 days off work due to LBP				
				Odds of reporting posture-related discomfort (reference: no discomfort) Previous 12 month LBP OR 4.40 (95% CI 2.72–7.10) Previous 7 day LBP OR 3.42 (95% CI 2.19–5.34)				
						12 month prevalence	Weekly prevalence	
				Police				
				Back pain only		73%	82%	
				Back and Leg pain/symptom		27%	18%	
				Truck/van				
				Back pain only		55%	57%	
				Back and Leg pain/symptom		45%	43%	
				Bus				
				Back pain only		53%	42%	
				Back and Leg pain/symptom		42%	27%	
				Leg pain/symptom only		-	5%	
				Taxi				
				Back pain only		32%	31%	
				Back and Leg pain/symptom		58%	54%	
				Leg pain/symptom only		-	15%	
				Total vibration dose–response trend for previous/current LBP (*p* = 0.037)				
						Previous LBP	Current LBP	
				8.6–15.0 years m^2^s^−4^ (n = 32):		OR 1.30 (95% CI 0.55–3.08)	OR = 0.89 (95% CI 0.36–2.22)	
				>15.0 years m^2^s^−4^ (n = 33):		OR 1.52 (95% CI 0.59–3.93)	OR = 1.31 (95% CI 0.53–3.23)	
				Posture score–response trend for previous/current LBP: (*p* = 0.013)				
						Previous LBP	Current LBP	
				0–6 points (n = 77):		Not reported	Not reported	
				7–12 points (n = 66)		OR 2.00 (95% CI 0.96–4.17)	OR 1.29 (95% CI 0.60–2.78)	
				>12 points (n = 46)		OR 2.04 (95% CI 0.91–4.58)	OR 1.95 (95% CI 0.86–4.39)	
Okunribido et al., 2007 [45]	♂/♁ drivers with at least one year in present job, or total of five years continuous bus driving experience (n = 61) ♂ = 58 ♁ = 3 Mean age: 47.6 ± 10.4 (19–64) years Mean weight: 84.9 ± 15.7 (58.6–129) kg Drivers with LBP (n = 36) Mean age: 48.1 ± 9.7 years Mean weight: 85.1 ± 13.3 kg Mean BMI: 28.5 ± 4.0 kg/m^2^ Mean height: 172.9 ± 7.49 cm Mean driving hours: 7.5 ± 1.4 (daily) Drivers without LBP (n = 25) Mean age: 46.8 ± 11.5 years Mean weight: 84.7 ± 19.1 kg Mean BMI: 28.1 ± 4.9 kg/m^2^ Mean height: 172.9 ± 9.82 cm Mean driving hours: 7.6 ± 1.8 (daily)	Bus, coach/asphalt and cobble surfaces	Self-assessment questionnaire previously employed in other research Tri-axial seat pad accelerometer (whole- body vibration meter 2.0)	Total of 23 drivers (38%) experience discomfort from sitting during driving, and 11 of those drivers report seats and bad back rest support Total of 42 drivers (69%) indicate discomfort from vibration Total of 36 drivers (59%) experience LBP during last 12 months and of those, 19 report current LBP within 7 days Drivers who report LBP are on average older and heavier Vibration dose value for moving on asphalt: Vibration dose values for single decker buses exceeds the European directive limit (safe daily exposure limit) on asphalt surfaces. Single decker: *x-axis 6.72 m/s^1.75^ y-axis 15.86 m/s^1.75^ z-axis 19.23 m/s^1.75^* Vibration dose value for moving on cobble: Vibration dose values for the mini-bus, double decker, and single decker buses exceeds the European directive limit (safe daily exposure limit). In particular, double decker and single decker buses (>15 m/s^1.75^), are associated with high vibration dose, indicating severe shock events on cobble surfaces Mini-bus: *x-axis 6.29 m/s^1.75^ y-axis 10.85 m/s^1.75^ z-axis 21.62 m/s^1.75^* Double decker: *x-axis 36.72 m/s^1.75^ y-axis38.53 m/s^1.75^ z-axis 38.87 m/s^1.75^* Single decker: *x-axis 10.12 m/s^1.75^ y-axis 27.04 m/s^1.75^ z-axis 37.60 m/s^1.75^*				67.5%
Rehman et al., 2018 [9]	Drivers with at least one year of driving experience, and a minimum travel of 35 h per/week (n = 377) Mean age: 39.7 ± 11.3 (18–67) years	Truck/long distance	ODI	Lower back disability more prevalent with advancement of age: LBP 18–27 years: 15% LBP 28–37 years: 56% LBP 38–47 years: 88% LBP 48–57 years: 71% LBP 58–67 years: 100%				67.5%
				Level of disability increased with greater travelling hours *p* < 0.001				
					Mild pain	Moderate pain	Severe pain	
				15–34 h/week	15%	2%	0%	
				35–64 h/week:	11%	5%	3%	
				65–84 h/week:	78%	6%	3%	
				85–104 h/week:	4%	71%	10%	
				105–124 h/week:	40%	30%	30%	
Rufai et al., 2015 [16]	♂ drivers with at least one year of driving experience, and drive for a minimum of five hours per day (n = 200) Mean age: 42.5 ± 11.1 (19–64) years Mean driving experience: 17.8 ± 7.9 (1–30) years Mean driving duration: 13.4 ± 6.3 (5–24) hours per day Drivers with LBP (n = 147) Drivers without LBP (n = 53) (Note: error noted in published Table 2)	Bus, truck, car/long distance	Modified NLBQ	LBP 74% (n = 147) Highest prevalence of LBP (59%) in bus drivers (n = 86) Gradual onset of LBP: 62% (n = 91) Mild severity of LBP: 21% (n = 31) Moderate severity of LBP: 49% (n = 72) 49% of participants with LBP (n = 72) ≥ 20 years driving experience 48% of participants (n = 70) history of >15 h driving per day Older drivers higher risk of developing LBP: 45–60 years: 0.18 times odds of having LBP (95% CI 0.40–0.71), *p* = 0.015 >60 years: 0.09 times odds of having LBP (95% CI 0.01–0.54), *p* = 0.010 Longer hours/day more vulnerable of developing LBP 10–15 h/day driving and LBP: 0.39 times odds of having LBP (95% CI 0.19–0.81), *p* = 0.011 Those who drive cars: 5.52 times odds of LBP occurrence (95% CI 1.55–19.64), *p* = 0.008				75%
Sangiamsak and Thetkathuek, 2021 [48]	♂ drivers, less than 1–15 years driving experience (n = 25) Terminal container shipment: short distance (n = 15) Seaport to urban logistics distribution: long distance (n = 10) Mean age: 38.5 ± 7.3 (20–60) years BMI: Healthy, normal n = 8 (32%) Overweight n = 6 (24%) Pre-obese n = 7 (28%) Obese n = 4 (16%) Work experience: <1–15 years	Truck/deep sea port road	NMQ (modified/translated into Thai language), Borg CR10 scale	LBP highest self-reported MSK symptom prevalence (72%), regardless of driving distance Neck: 32% Knees: 28% Short distance: Lower back: 44% Hip/thigh: 20% Knees/ankle/feet: 16% Long distance: Lower back: 28% Neck: 24% Knees: 12% A total of 88% of drivers experience MSK symptoms over last 12 months: Short distance: 52% Long distance: 36% Prevalence of neck pain higher in long distance truck drivers than short distance truck drivers, *p* = 0.028 Lower back perceived discomfort (2.4), neck (1.44), and knee (1.28)				75%
Selvam and Arun, 2016 [43]	♂ drivers working ≥ 8 h/day, and 5–10 years of driving experience with VAS score of ≥7 (n = 10) Age range: 25–40 years	Bus/not stated	Scapular protraction measurement VAS	Scapular protraction is greater with hands on hips than at rest, or at 90° abduction for right and left side. VAS for mechanical neck pain when hands on hips: Right: 7.2 Left: 6.9				50%
Senthanar and Bigelow, 2018 [2]	♂ drivers who have driven for a minimum of six months and ≥18 years old (n= 107) With musculoskeletal pain: (n = 61) Mean age: 45 ± 10.0 (23–65) years Mean work experience: 6.5 ± 10.3 (3–40) years Mean driving hours: 47.2 ± 21.4 (1–100) (weekly) Without musculoskeletal pain: (n = 46) Mean age: 53 ± 10.1 (26–67) years Mean work experience: 21.2 ± 14.5 (3–50) years Mean driving hours: 44.9 ± 25.3 (4–92) (weekly)	Truck (long haul)/highways	Questionnaire developed by OHS professionals working directly with long-haul truck drivers in transport sector	A total of 57% complain of MSK pain and discomfort (n = 61) Prevalence of MSK Pain and discomfort: Lower back: 80% Shoulders: 54% Wrist/hands: 44% Legs/feet: 41% Upper back: 39% MSK pain and discomfort driving >7 h: OR 1.12 (95% CI 1.01–1.24) Factors predicting MSK pain and discomfort: *Organisational:* job control (*p* ≤ 0.01); safety equipment availability (*p* ≤ 0.01); and management involved in injury (*p* ≤ 0.05) *Physical:* level of risk (*p* ≤ 0.05); perceptions of jobs/tasks (*p* ≤ 0.01)				75%
Szeto and Lam, 2007 [3]	♂/♁ drivers (n = 481) ♂: 404, ♁: 77 Mean age: ♂: 47.5 ± 7.4 years ♁: 46.4 ± 5.3 years Mean weight: ♂: 71.2 ± 10.5 kg ♁: 58.8 ± 6.7 kg Mean BMI: ♂ 25.2 ± 3.4 kg/m^2^ ♁ 23.6 ± 2.7 kg/m^2^ Mean height: ♂167.8 ± 5.9 cm ♁ 158.1 ± 5.5 cm Mean work experience: ♂ 13.0 ± 8.9 years ♁ 8.6 ± 3.5 years Mean driving hours: ♂: 10.0 ± 1.0 per day ♁: 9.8 ± 0.7 per day	Bus (double decker)/urban, city	NMQ (Chinese version)	No discomfort: 7% (n = 35) One area of discomfort: 23% (n = 110) >2 areas of discomfort: 64% (n = 306) Younger age groups (<40 years) and those with less work experience have increased discomfort Duration of pain and discomfort: Total of 5+ years: 35–40% Between 1–4 years: 50% LBP and neck pain highest 12 month prevalence 4.5–5.0/10, indicating moderate severity Self-perceived occupational risk factors: Prolonged sitting (n = 355) Driver seat mismatch (n = 196) Steering wheel tightness (n = 151) Mechanical vibration (n = 116) Sex (reference: males) Neck pain OR 1.78 (95% CI 1.03–3.06), *p* = 0.038 Shoulder pain OR 1.91 (95% CI 1.10–3.31), *p* = 0.022 Knee/thigh pain OR 1.70 (95% CI 1.01–2.86), *p* = 0.047 Prolonged sitting LBP: OR 3.71 (95% CI 2.40–5.74), *p* < 0.001 Driver seat mismatch Neck pain: OR 1.56 (95% CI 1.06–2.30), *p* = 0.026 Shoulder pain: OR 2.21 (95% CI 1.49–3.26), *p* ≤ 0.001 Working years (>16 years) Shoulder pain: OR 2.4 (95% CI 1.37–4.49), *p* = 0.003 Gear box control Thigh/knee pain: OR 2.74 (95% CI 1.14–6.60), *p* = 0.025				87.5%
Tamrin et al. 2007 [49]	Drivers (n = 760) Mean age: 43.0 ± 8.64 years Mean duration of work: 96.4 ± 3.3 months Mean driving hours: 10.5 ± 0.1 (day) Mean driving hours: 60.5 ± 0.7 (weekly)	Smooth and rough road surface in urban, suburban, rural, residential, and industrial areas	NMQ	Lower back pain: 60% (n = 459) Neck: 52% (n = 392) Upper back: 41% (n = 309) Shoulder: 35% (n = 269) No posture significantly associated with LBP Duration employment as bus driver (96.38 ± 3.30 months): OR 1.00 (95% CI 1.00–1.01) Perception exposure to vibration: OR 1.94 (95% CI 1.39–2.72) Steering wheel adjustability: OR 1.52 (95% CI 1.10–2.10)				50%
Wang et al., 2017 [47]	Drivers with at least one year experience driving, and work 40 h per week (n = 719) ♂ (n = 694) ♁: (n = 25) Mean age: 40.1 ± 5.8 (26–54) years Mean BMI: 24.6 ± 2.9 kg/m^2^ Mean driving hours: 10.8 ± 2.3 (daily) Mean driving experience: 7.7 ± 3.5 (1–20) years	Taxi, not stated	MDQ/NMQ	Prevalence of LBP in 12 months: 54% (n = 388) (95% CI 50.00–58.00) Risk of reporting LBP increases with: Higher BMI: BMI 24–28 kg/m^2^: OR 1.90 (95% CI 1.20–3.00), *p* < 0.05 BMI ≥ 28 kg/m^2^: OR 1.80 (95% CI 1.10–2.80), *p* < 0.01 Daily driving duration: 8–12 h: OR 2.20 (95% CI 1.30–3.70), *p* < 0.01 ≥12 h: OR 2.30 (95% CI 1.60–3.30), *p* < 0.001 More work years: Work years group ≥ 10 years: OR 1.70 (95% CI 1.20–2.40), *p* < 0.01 Night shifts: OR 2.30 (95% CI 1.70–3.20), *p* < 0.001 Odds of LBP decrease with; More rest days: OR 0.80 (95% CI 0.70–0.90), *p* < 0.01 Longer sleep duration: OR 0.70 (95% CI 0.50–0.80), *p* < 0.05 More physical activities: OR 0.50 (95% CI 0.30–0.70), *p* < 0.001				75%

CAS: critical appraisal score; BMI: body mass index; FFI: Foot Function Index; LBP: low back pain; MSK: musculoskeletal; MDLB: modified Nordic low back; MDQ: modified Delphi questionnaire; NPRS: numerical rating pain scale; NLBQ: Nordic LBP questionnaire; NMQ: Nordic musculoskeletal questionnaire; NLBPQ: Nordic low back pain questionnaire; ODI: Oswestry Disability Index; OLBDI: Oswestry lower back Disability Index; RMDQ: Roland–Morris disability questionnaire; VAS: visual analogue scale; WRMSD: work-related musculoskeletal disorders.

## Data Availability

All data (i.e., individual appraisal scores) will be included in the final submission.

## Supplementary Materials

The following supporting information can be downloaded at: https://www.mdpi.com/article/10.3390/ijerph19116837/s1, Supplemental Table S1: Excluded studies with reason. References [73,74,75,76,77,78,79,80,81,82,83,84,85,86,87,88,89,90,91,92,93] are cited in the Supplementary Materials.
