# Supplementary material for: Musculoskeletal Disorders Associated with Occupational Driving: A Systematic Review Spanning 2006–2021
Source: Int J Environ Res Public Health. 2022 Jun 2;19(11):6837. doi: 10.3390/ijerph19116837 (PMC9180502; doi:10.3390/ijerph19116837)
Supplement: Supplementary file 1 [file ijerph-19-06837-s001.zip › ijerph-1681033-supplementary.pdf]

Supplemental Table S1. Excluded studies with reason

Reason for Exclusion	References
Injuries not related to driving	1-7
Accident/Crash related injuries	8-9
Injury not clearly defined (e.g Discomfort)	10-19
Vehicles other than road vehicles included in the data (e.g Crane or train)	20-25 (27)
Pre-existing injuries	27-29
Altered driving surface (dirt racing track)	30

Excluded References:

1. Anderson, N.J.; Smith, C.K.; Byrd, J.L. Work-related injury factors and safety climate perception in truck drivers. *American Journal of Industrial Medicine* **2017**, *60*, 711-723, doi:10.1002/ajim.22737.
2. Anderson, C.K. Relationship between aerobic capacity, injury risk and tenure for new-hire delivery drivers. *Ergonomics* **2010**, *53*, 1395-1401, doi:10.1080/00140139.2010.524252.
3. Bréder, V.F.; Dantas, E.H.M.; Silva, M.A.G. Low Back Pain And Psychosocial Factors Among Bus Drivers. *Fitness & Performance Journal (Online Edition)* **2006**, *5*, 36-44.
4. Chandler, M.D.; Bunn, T.L.; Slavova, S. Narrative and quantitative analyses of workers' compensation-covered injuries in short-haul vs. long-haul trucking. *International Journal of Injury Control and Safety Promotion* **2017**, *24*, 120-130, doi:10.1080/17457300.2016.1170041.
5. Combs, B.; Heaton, K.; Raju, D.; Vance, D.E.; Sieber, W.K. A Descriptive Study of Musculoskeletal Injuries in Long-Haul Truck Drivers: A NIOSH National Survey. *Workplace Health & Safety* **2018**, *66*, 475-481, doi:10.1177/2165079917750935. [17]
6. Lukman, K.A.; Jeffree, M.S.; Rampal, K.G. Lower back pain and its association with whole-body vibration and manual materials handling among commercial drivers in Sabah. *International Journal of Occupational Safety and Ergonomics* **2019**, *25*, 8-16, doi:10.1080/10803548.2017.1388571.
7. Sekkay, F.; Imbeau, D.; Chinniah, Y.; Dubé, P.-A.; de Marcellis-Warin, N.; Beauregard, N.; Trépanier, M. Risk factors associated with self-reported musculoskeletal pain among short and long distance industrial gas delivery truck drivers. *Applied Ergonomics* **2018**, *72*, 69-87, doi:10.1016/j.apergo.2018.05.005.
8. Anderson, J.; Hernandez, S. Roadway classifications and the accident injury severities of heavy-vehicle drivers. *Analytic Methods in Accident Research* **2017**, *15*, 17-28, doi:10.1016/j.amar.2017.04.002.
9. Attarchi, M.S.; Dehghan, F.; Seyedmehdi, S.M.; Mohammadi, S. Traffic accidents and related injuries in Iranian professional drivers. *Journal of Public Health-Heidelberg* **2012**, *20*, 499-503, doi:10.1007/s10389-011-0474-7.
10. Apostolopoulos, Y.; Sönmez, S.; Shattell, M.M.; Belzer, M. Worksite-induced morbidities among truck drivers in the United States. *AAOHN Journal* **2010**, *58*, 285-296, doi:10.3928/08910162-20100625-01.
11. Araujo, A.V.; Arcanjo, G.S.; Fernandes, H.; Arcanjo, G.S. Ergonomic work analysis: A case study of bus drivers in the private collective transportation sector. *Work-a Journal of Prevention Assessment & Rehabilitation* **2018**, *60*, 41-47, doi:10.3233/wor-182718.
12. Cardoso, M.; Girouard, M.; McKinnon, C.; Callaghan, J.P.; Albert, W.J. Quantifying the postural demands of patrol officers: a field study. *International Journal of Occupational Safety and Ergonomics* **2017**, *23*, 185-197, doi:10.1080/10803548.2016.1249729.
13. Cardoso, M.; Girouard, M.; Callaghan, J.P.; Albert, W.J. An ergonomic evaluation of city police officers: an analysis of perceived discomfort within patrol duties. *International Journal of Occupational Safety and Ergonomics* **2017**, *23*, 175-184, doi:10.1080/10803548.2016.1249728.
14. Cardoso, M.; Fulton, F.; McKinnon, C.; Callaghan, J.P.; Johnson, M.J.; Albert, W.J. Ergonomic evaluation of a new truck seat design: a field study. *International Journal of Occupational Safety and Ergonomics* **2019**, *25*, 331-343, doi:10.1080/10803548.2017.1348056.

15. Donnelly, C.J.; Callaghan, J.P.; Durkin, J.L. The Effect of an Active Lumbar System on the Seating Comfort of Officers in Police Fleet Vehicles. *International Journal of Occupational Safety and Ergonomics* **2009**, *15*, 295-307, doi:10.1080/10803548.2009.11076809.
16. Gil de Alcantara, V.C.; Costa Rosa Andrade Silva, R.M.; Ramos Pereira, E.; Melo da Silva, D.; Pinto Flores, I. The experience in traffic and its effects on the health of bus drivers: a phenomenological descriptive study. *Revista de Enfermagem Referência* **2019**, *4*, 21-29, doi:10.12707/RIV19049.
17. Jensen, A.; Kaerlev, L.; Tuchsen, F.; Hannerz, H.; Dahl, S.; Nielsen, P.S.; Olsen, J. Locomotor diseases among male long-haul truck drivers and other professional drivers. *International Archives of Occupational and Environmental Health* **2008**, *81*, 821-827, doi:10.1007/s00420-007-0270-4.
18. Lewis, C.A.; Johnson, P.W. Whole-body vibration exposure in metropolitan bus drivers. *Occupational Medicine-Oxford* **2012**, *62*, 519-524, doi:10.1093/occmed/kqs096.
19. Serrano-Fernandez, M.J.; Boada-Grau, J.; Robert-Sentis, L.; Vigil-Colet, A. Predictive variables for musculoskeletal problems in professional drivers. *Journal of Transport & Health* **2019**, *14*, doi:10.1016/j.jth.2019.100576.
20. Bovenzi, M.; Schust, M.; Menzel, G.; Hofmann, J.; Hinz, B. A cohort study of sciatic pain and measures of internal spinal load in professional drivers...5th International Conference on Whole Body Vibration Injuries, Amsterdam, The Netherlands, 5-7 June 2013. *Ergonomics* **2015**, *58*, 1088-1102, doi:10.1080/00140139.2014.943302.
21. Bovenzi, M.; Schust, M.; Mauro, M. An overview of low back pain and occupational exposures to whole-body vibration and mechanical shocks. *Medicina Del Lavoro* **2017**, *108*, 419-433, doi:10.23749/mdl.v108i6.6639.
22. Bovenzi, M. Metrics of whole-body vibration and exposure-response relationship for low back pain in professional drivers: a prospective cohort study. *International Archives of Occupational and Environmental Health* **2009**, *82*, 893-917, doi:10.1007/s00420-008-0376-3.
23. Bovenzi, M. A Longitudinal Study of Low Back Pain and Daily Vibration Exposure in Professional Drivers. *Industrial Health* **2010**, *48*, 584-595.
24. Koutras, C.; Antoniou, S.A.; Jager, M.; Heep, H. Acute Injuries Sustained by Racing Drivers: A Cross-Sectional Study. *Acta Orthopaedica Belgica* **2017**, *83*, 512-520.
25. Lee, J.W.; Lim, Y.H.; Won, Y.H.; Kim, D.H. Effect of gel seat cushion on chronic low back pain in occupational drivers A double-blind randomized controlled trial. *Medicine* **2018**, *97*, doi:10.1097/md.0000000000012598.
26. Rugulies, R.; Krause, N. Effort-reward imbalance and incidence of low back and neck injuries in San Francisco transit operators. *Occupational and Environmental Medicine* **2008**, *65*, 525-533, doi:10.1136/oem.2007.035188.
27. Bovenzi, M. A prospective cohort study of neck and shoulder pain in professional drivers...5th International Conference on Whole Body Vibration Injuries, Amsterdam, The Netherlands, 5-7 June 2013. *Ergonomics* **2015**, *58*, 1103-1116, doi:10.1080/00140139.2014.935487.
28. Ghasemi, M.; Khoshakhlagh, A.H.; Ghanjal, A.; Yazdanirad, S.; Laal, F. The impacts of rest breaks and stretching exercises on lower back pain among commercial truck drivers in Iran. *International Journal of Occupational Safety and Ergonomics* **2020**, *26*, 662-669, doi:10.1080/10803548.2018.1459093.
29. Zack, O.; Levin, R.; Krakov, A.; Finestone, A.S.; Moshe, S. The relationship between low back pain and professional driving in young military recruits. *BMC Musculoskeletal Disorders* **2018**, *19*, N.PAG-N.PAG, doi:10.1186/s12891-018-2037-3.
30. Ebben, W.P.; Suchomel, T.J. Physical Demands, Injuries, and Conditioning Practices of Stock Car Drivers. *Journal of Strength and Conditioning Research* **2012**, *26*, 1188-1198, doi:10.1519/JSC.0b013e31822d5306.
